# Expression and localisation of MUC1 modified with sialylated core-2 *O*-glycans in mucoepidermoid carcinoma

**DOI:** 10.1038/s41598-023-32597-2

**Published:** 2023-04-08

**Authors:** Takanori Sugiura, Kazuhiko Hashimoto, Kazutaka Kikuta, Ukei Anazawa, Takeshi Nomura, Akihiko Kameyama

**Affiliations:** 1 Department of Oral Oncology, Oral and Maxillofacial Surgery, Ichikawa General Hospital, Tokyo Dental College, 5-11-13 Sugano, Ichikawa-Shi, Chiba 272-8513 Japan; 2 Department of Pathology and Laboratory Medicine, Ichikawa General Hospital, Tokyo Dental College, 5-11-13 Sugano, Ichikawa-Shi, Chiba 272-8513 Japan; 3 Department of Musculoskeletal Oncology and Orthopaedic Surgery, Tochigi Cancer Center, 4-9-13 Yohnan, Utsunomiya, Tochigi 320-0834 Japan; 4 Department of Orthopaedic Surgery, Ichikawa General Hospital, Tokyo Dental College, 5-11-13 Sugano, Ichikawa-Shi, Chiba 272-8513 Japan; 5 Oral Cancer Center, Tokyo Dental College, 5-11-13 Sugano, Ichikawa-Shi, Chiba 272-8513 Japan; 6 Cellular and Molecular Biotechnology Research Institute, National Institute of Advanced Industrial Science and Technology (AIST), 1-1-1 Higashi, Tsukuba, Ibaraki 305-8565 Japan

**Keywords:** Biochemistry, Biotechnology, Cancer, Chemical biology, Drug discovery, Biomarkers, Medical research

## Abstract

Mucoepidermoid carcinoma (MEC) is the most frequent of the rare salivary gland malignancies. We previously reported high expression of Mucin 1 (MUC1) modified with sialylated core-2 *O*-glycans in MEC by using tissue homogenates. In this study, we characterised glycan structures of MEC and identified the localisation of cells expressing these distinctive glycans on MUC1. Mucins were extracted from the frozen tissues of three patients with MEC, and normal salivary glands (NSGs) extracted from seven patients, separated by supported molecular matrix electrophoresis (SMME) and the membranes stained with various lectins. In addition, formalin-fixed, paraffin-embedded sections from three patients with MEC were subjected to immunohistochemistry (IHC) with various monoclonal antibodies and analysed for *C2GnT-1* expression by in situ hybridisation (ISH). Lectin blotting of the SMME membranes revealed that glycans on MUC1 from MEC samples contained α2,3-linked sialic acid. In IHC, MUC1 was diffusely detected at MEC-affected regions but was specifically detected at apical membranes in NSGs. ISH showed that *C2GnT-1* was expressed at the MUC1-positive in MEC-affected regions but not in the NSG. MEC cells produced MUC1 modified with α2,3-linked sialic acid-containing core-2 *O-*glycans. MUC1 containing these glycans deserves further study as a new potential diagnostic marker of MEC.

## Introduction

Salivary gland tumours account for approximately 1% of all tumours and 3–6% of head and neck tumours^1^. The histopathology of salivary gland tumours is highly variable and broadly diverse, with 33 histological types classified by the 2017 WHO classification^2^. Mucoepidermoid carcinoma (MEC) is the most frequent of the rare salivary gland malignancies^2–4^. Surgery is the first-line treatment for salivary gland tumours, including MEC, with no established radiotherapy or chemotherapy, and there are no useful biomarkers for diagnoses or treatments. MEC has a distinct feature of producing mucus and abnormally expressing Mucin 1 (MUC1), a type of mucin that is a heavily glycosylated, high-molecular-weight glycoprotein^4–6^. MUC1 is modified with a large number of *O*-glycans that link to hydroxy groups of Ser or Thr residues of the peptide backbone through glycosidic linkage. Alterations from glycosylation are often observed in tumours. MUC1 glycans should be sensitively reflected by malignant transformation due to their heavily glycosylated property. The serum tumour marker, CA19-9 and the mesothelial cancer tumour marker, sialylated HEG1, are well-known examples for glycan alteration of MUC1 accompanied by malignant transformation^7–9^.

Previously, we found that MEC produced MUC1 distinctively modified by sialylated core-2 *O*-glycans (GlcNAcβ1-6(Galβ1-3)GalNAcαSer/Thr)^10^. However, it remains unclear which cells in MEC tissues produce such characteristic modified MUC1. In addition, the type of sialic acid linkage to the core-2 *O-*glycan also has not been determined.

The purpose of this study was to elucidate the structure of these distinctive glycans and determine the localisation of cells expressing them on MUC1 to develop a novel biomarker candidate for diagnosis of MEC. Our study identified localisation of cells expressing MUC1 with these glycans in formalin-fixed, paraffin-embedded (FFPE) sections of MEC by immunohistochemistry (IHC) and in situ hybridisation (ISH). In addition, the structures of the distinctive glycans on MUC1 were elucidated by lectin blottings of the supported molecular matrix electrophoresis (SMME) membranes that separate mucins of MEC homogenates.

## Result

### Glycans analysis by SMME lectin blotting

**Figure 1:**
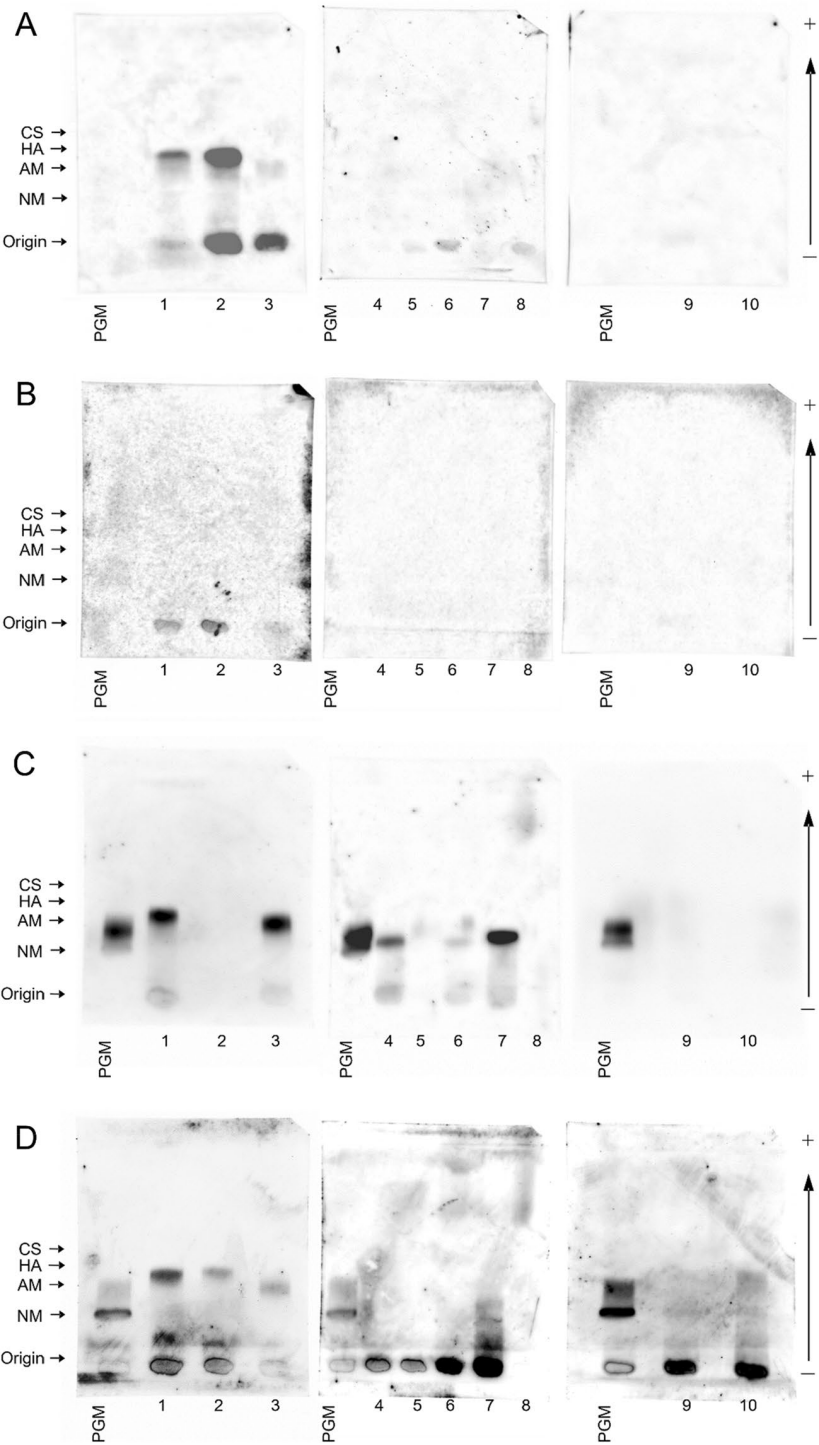
Lectin blotting of the SMME membranes separating salivary gland homogenates. (**A**) MAL-II; (**B**) SSA; (**C**) BC2LCN; (**D**) AAL. Sample numbers 1–3: MEC and 4–10: NSGs. Sample properties are summarised in Table 3. Partially purified porcine gastric mucin (PGM) was used as a reference mixture of chondroitin sulphate (CS), hyaluronic acid (HA), acidic mucin (AM) and neutral mucin (NM). The migrating positions of these components of PGM were estimated from a previous report^19^. The original, unprocessed versions of full-length lectin blotting images are included in the supplemental (Supplemental Fig. S1).

In SMME lectin blotting, comparisons were made between MEC and NSG. Mucins extracted from these samples were separated by SMME, and then the mucins on the SMME membranes were stained with lectins recognising sialic acid residues or fucose residues. These residues attach to the non-reducing end of the glycans and are composed of antigenic determinants, such as Lewis blood-type antigens and tumour-associated glycan antigens, including CA19-9. Specificities of the lectins used in this study are summarised in Table 1. The mucins in all three patients (1–3) with MEC were stained with MAL-II (Fig. 1A) but not with SSA (Fig. 1B). MAL-II and SSA did not stain any bands of NSGs (4–10) except for some origin spots. In contrast, no mucins were modified with sialic acids in NSGs.

**Table 1:**
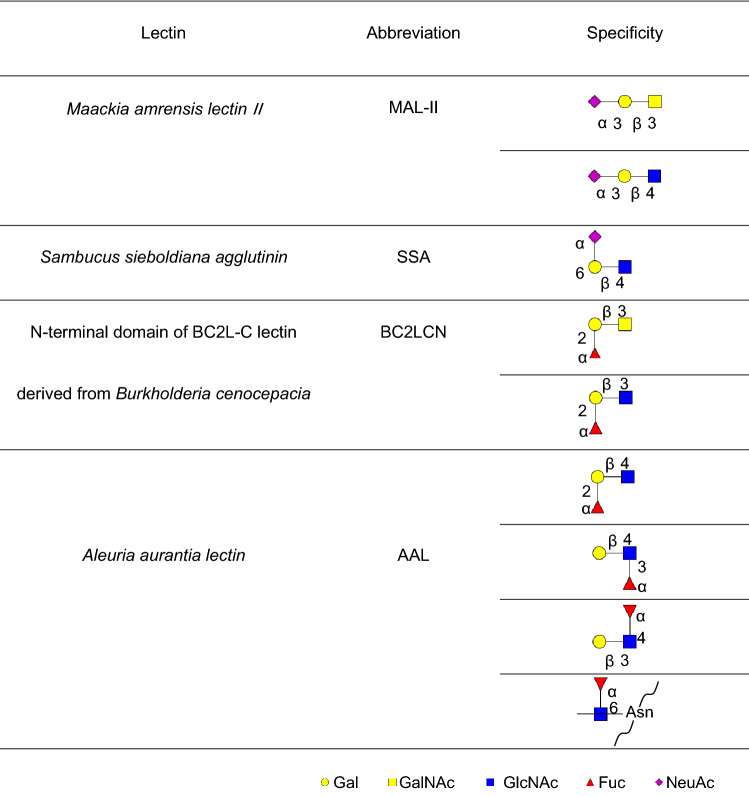
Summary of the lectins used in this study.

The fucose-binding lectins, BC2LCN and AAL, stained some mucin bands, including mucins of PGM regardless of MEC or NSGs (Fig. 1C,D). Mucins in two patients (1, 2) with MEC and three patients (4, 6, 7) with NSGs were stained with BC2LCN (Fig. 1C). Mucins in all patients (1–3) with MEC and three patients (7, 9, 10) with NSGs were stained with AAL (Fig. 1D). In addition, AAL stained some origin spots (1–7, 9, 10) (Fig. 1D).

### Localisation of modified MUC 1 with sialylated core-2 *O*-glycans

**Figure 2:**
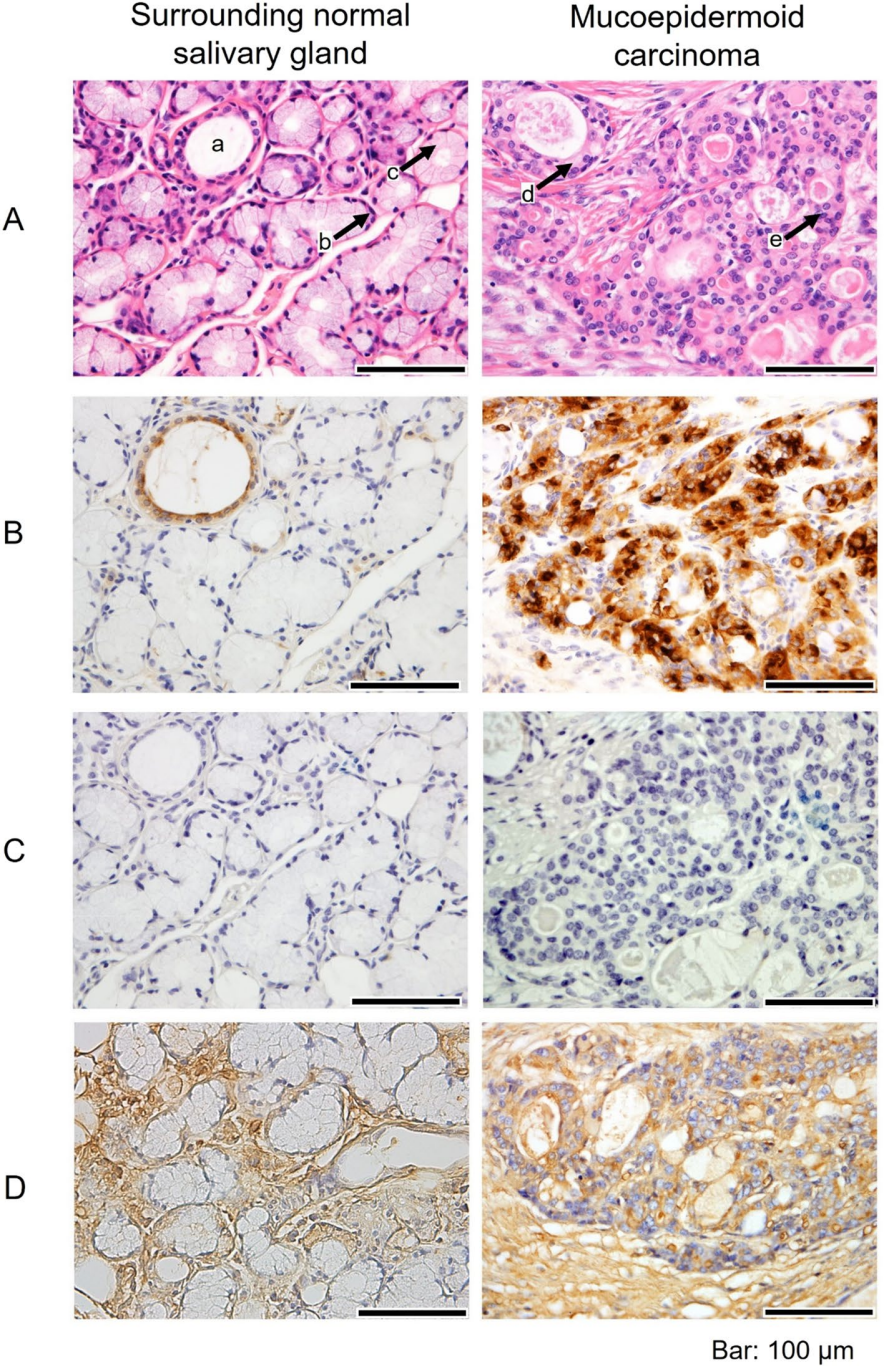
Comparison of the expression patterns of MUC1 and sialoglycans between MEC and its surrounding normal salivary gland. The representative images (original magnification, × 400) of H&E staining (**A**), staining with anti-MUC1 antibody (**B**), staining with anti-Sialyl-Tn antibody (**C**) and staining with MAL-II (**D**) are shown. Ducts (a), serous acini (b) and mucous acini (c) are indicated by H&E staining of the normal salivary gland, and mucous cells (d) and non-mucous cells (e) are indicated in H&E staining of the MEC (**A**). Scale bars indicate 100 µm.

**Figure 3:**
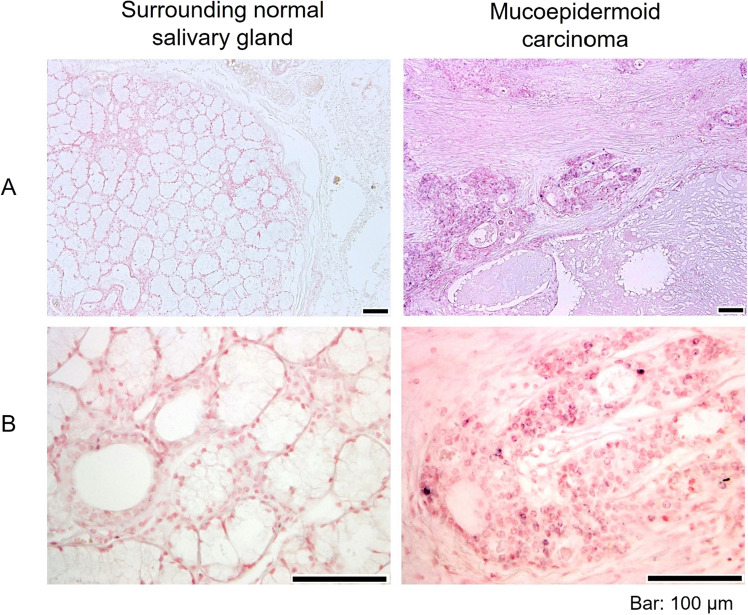
Comparison of the expression patterns of C2GnT-1 between MEC and its surrounding normal salivary gland. The representative images (original magnification, × 100 (**A**) and × 400 (**B**)) of ISH of *C2GnT-1* are shown. Scale bars indicate 100 µm.

In IHC and ISH, comparisons were made between MEC and the surrounding NSGs. On HE staining, the salivary glands around the MEC were histologically normal and composed of ducts (a), serous acini (b) and mucous acini (c), as shown in Fig. 2A. MEC was marked by mucous (d) and non-mucous cells (e) (epidermoid and intermediate cells, respectively). Mucous and non-mucous cells were histologically and morphologically distinct. Mucous cells have rich cytoplasm and small nucleus are usually compressed and located near the periphery of the cell. Intermediate cells have a small, darkly staining nucleus and scant cytoplasm. Epidermoid cells have intercellular bridges and resemblance to squamous cell epithelium^11^. The MEC areas were compared with the surrounding NSGs by staining with anti-MUC1 antibody, anti-sialyl-Tn antibody that is a well-known tumour-associated antigen and MAL-II. In this study, immunohistochemical evaluations were considered to be positive when > 5% of cells were stained and are summarised in Table 2 and Supplemental Table 1. Representative staining images are shown in Figs. 2 and 3. The mucous and non-mucous cells in all patients with MEC were MUC1-positive, whereas only ducts were MUC1-positive in 2/3 patients of the surrounding normal salivary gland. The mucus acini and the serous acini of the normal tissue were not stained by anti-MUC1 antibody (Fig. 2B). MUC1 expression was confined to the apical membrane of ducts in the normal tissues but diffusive in mucous and non-mucous cells of MEC. The mucous and non-mucous cells in all patients with MEC were MAL-II-positive, and ducts, serous acini and mucous acini were both MAL-II-positive and MUC1-positive in all patients of the surrounding normal salivary gland (Fig. 2D).

**Table 2:** MUC1, Sialyl-Tn, MAL-II and C2GnT-1 expressions in normal salivary glands and mucoepidermoid carcinomas.

	Surrounding normal salivary gland			Mucoepidermoid carcinoma	
	Mucous acini	Serous acini	Ducts	Mucous cells	Non-mucous cells
MUC1	0/3	0/3	2/3	3/3	3/3
sialyl-Tn	0/3	0/3	0/3	1/3	1/3
MAL-II	3/3	3/3	3/3	3/3	3/3
C2GnT-1	0/3	0/3	0/3	3/3	3/3

**Figure 4:**
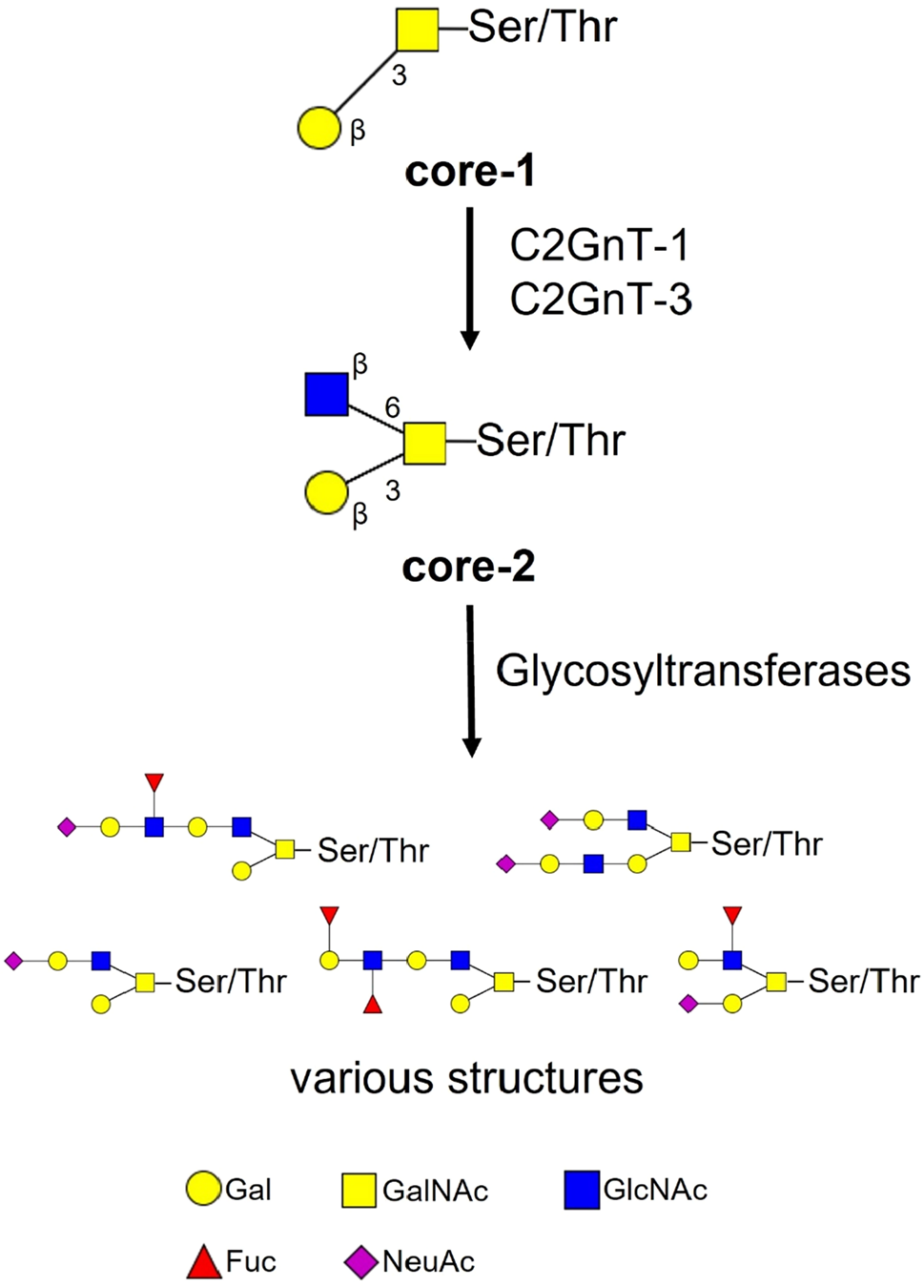
C2GnTs are the key enzyme for core-2 *O*-glycan biosynthesis. C2GnTs transfer a GlcNAc residue to the 6-position of GalNAc in core-1 disaccharide to form a core-2 trisaccharide. The core-2 trisaccharide is further modified by glycosyltransferases, including sialyltransferases and fucosyltransferases to turn various meaningful structures, such as the sialyl Lewis type, into a determinant.

We then evaluated the expression of *C2GnT-1*, the gene responsible for β1,6 *N*-acetylglucosaminyl transferase that is a key enzyme for biosynthesis of the core-2 *O*-glycans (Fig. 4).

ISH showed that *C2GnT-1* expression was not detected in the surrounding normal tissues in any of the three patients, whereas it was detected in all patients with MEC (Fig. 3).

Detailed IHC and ISH staining results are in Supplemental Figs. S2–S7.

## Discussion

Sialylation and fucosylation at the non-reducing end of glycans often form meaningful glycan determinants, such as blood-type antigens and tumour-associated glycan antigens. Alterations of these glycosylations have been reported in malignant transformation^12–14^. Increased sialylation is generally relevant to malignant tumours and promotes invasiveness and metastasis, leading to cancer progression and poor prognosis^15,16^. We previously found that sialylation on mucins increased in MEC. In this study, lectin blotting using MAL-II revealed that the mucins in MEC tissues were distinctively modified with sialic acids through an α2,3-linkage but not through an α2,6-linkage. Therefore, the glycans were found to be sialylated at the 3-position of the Gal residues. BC2LCN specifically recognises Fucα1-2Galβ1–3 structures that are synthesised in human salivary glands by the product of FUT2, a gene responsible for secretion. Approximately 20% of the Japanese population are non-secretors who cannot make the Fucα1-2Galβ1-3 structures due to inactivation of the FUT2 product^17^. In this experiment, 5 among 10 samples were BC2LCN negative and considered to be of non-secretor origin. AAL broadly recognises fucose residues, including Lewis fucose (Fucα1-3/4GlcNAc) and core fucose (Fucα1-6GlcNAc) of N-linked glycans. AAL stained all three patients (1–3) with MEC and any bands of NSGs (7, 9, 10) (Fig. 1D). Thus, these results suggested that glycans of mucins in MEC tissues had Lewis fucose and also had Fucα1-2Galβ1-3 in the secretors. In addition, all origin spots except for sample 8 were also stained with AAL. During the SMME with the 0.1 M pyridine-formic acid buffer (pH 4.0), many proteins with pI values ≥ 4.0 do not migrate from the origin. AAL might bind fucosylated N-linked glycans on these proteins. The α2,3-linked sialylation competes with α1,2 fucosylation at the same Gal that forms type-H antigen. BC2LCN that binds the type-H antigen, however, stained the MUC1 bands (samples 1 and 3) that were also stained with MAL-II (Fig. 1C). This finding implies that plural Gal residues were present in the individual glycans, and they are likely to occur in core-2 glycans that have a branching structure. Tandem mass spectrometry of the sialylated glycans in our previous study also suggested that the glycans were core-2 glycans. *O*-glycans in humans are classified into four types: cores 1–4 based on a di- or trisaccharide structure at the reducing end^18^. Among them, core-2 glycans are known to be elongated to form the glycan antigens, such as sialyl Lewis^X^ and sialyl Lewis^a^^19^. These glycan antigens on mucins and glycolipids have been used as serum tumour markers for several decades. In addition, sialyl Lewis^X^ and sialyl Lewis^a^ are also known to be the ligands of selectins that are a family of cell-adhesion molecules expressed on endothelial cells, leukocytes and platelets. These glycans on tumour cells have been implicated in tumour cell metastasis through adhesion to the vascular endothelium^20^. The mRNA of *C2GnT-1*, the gene responsible for core-2 trisaccharide synthesis, increases in pancreatic cancer and lung cancer, whereas it decreases in breast cancer^21^. In this study, the location of cells expressing C2GnT-1 mRNA overlapped with MUC1-positive areas in MEC. These results suggested that MUC1-positive cells in MEC also synthesise core-2 *O-*glycans. This possibility would support the idea that these cells express MUC1 modified with core-2 glycans. It has been reported that core-2 *O-*glycans suppress the immune function of natural killer (NK) cells, contributing to tumour cells to evade the host’s cancer-cell elimination mechanism^22^. The expression of MUC1 with core-2 *O-*glycans in MEC may be related to the poor prognosis of MEC.

In normal human tissues, membrane-bound MUC1 is known to be expressed at the apical surface of glandular or luminal epithelial cells, such as the lung, mammary gland, stomach and salivary glands^5, 13,23^. We confirmed restricted expression of MUC1 at the apical membranes in the surrounding normal tissue of MEC in this study. In contrast, diffuse staining was observed in the MEC. These results are consistent with those of previous reports^5,24^. Therefore, MUC1 with sialylated core-2 glycans is a potential biomarker for MEC in serum or saliva. However, it is not known whether MUC1 modified with this glycan can be detected in serum or saliva, so additional research is needed. In addition, whether or not MUC1 is localised in mucous cells and non-mucous cells of MEC is unknown and needs to be investigated.

Expression of sialyl-Tn, a well-known tumour-associated glycan antigen, was also evaluated and compared with expression of MUC1. Sialyl-Tn was only detected in one-third of the patients with MEC (Fig. 2C). This finding was consistent with our previous data showing that sialyl-Tn was rarely detected by mass spectrometry of the glycans from MUC1 separated by SMME of the MEC homogenate [our unpublished data]. The mass spectral data showed that sialylated core-2 *O-*glycans were major species of glycans on MUC1. Furthermore, we stained the sections with MAL-II to reveal the locations of α2,3-linked sialic acid residues. In this study, the location of cells expressing MAL-II overlapped with MUC1-positive areas in MEC. These results suggested that MUC1-positive cells in MEC also have α2,3-linked sialic acid residues. This possibility would support the idea that these cells express MUC1 modified with core-2 glycans containing α2,3-linked sialic acid.

One limitation of this study was that only three samples from patients with MEC were used. In addition, the MECs were from minor salivary glands, whereas NSGs include the major and minor salivary glands. However, all three patients with MEC showed similar results. Additionally, there was a clear difference in the results between MEC and NSGs. Given these differences, it would be worthwhile to increase the number of samples and collection of additional data in the future. Another limitation of this study was that the SMME used tissue homogenates. Therefore, an unknown effect of a tissue other than MEC and NSGs could not be completely excluded. However, results of IHC and ISH were considered to be acceptable to support the results of lectin blotting on the SMME membrane.

These results suggest that MUC1 modified with core-2 *O-*glycans containing α2,3-linked sialic acid is expressed in mucous cells and non-mucous cells of MEC.

## Materials and methods

### Normal and MEC samples

Mucins extracted from the frozen tissues of three patients with MEC and normal salivary glands (NSGs) extracted from seven patients (five submandibular, one sublingual and one minor salivary glands) were used (Table 3). All patients were diagnosed by an experienced oral pathologist, and all MECs were low grade.

**Table 3:** Summary of salivary glands used in this study.

Mucoepidermoid carcinoma			Normal salivary glands		
Sample number	Age	Location	Sample number	Age	Location
1	38	Minor salivary gland	4	90	Submandibular gland
2	39	Minor salivary gland	5	68	Submandibular gland
3	52	Minor salivary gland	6	57	Submandibular gland
			7	41	Submandibular gland
			8	55	Submandibular gland
			9	51	Sublingual gland
			10	87	Minor salivary gland

This research was approved by the Tokyo Dental College Ichikawa General Hospital Ethics Review Committee (I16-74) and the ethics committee of the National Institute of Advanced Industrial Science and Technology (AIST). Written informed consent was obtained from all patients. All procedures and methods were performed following the relevant guidelines and regulations, including the Declaration of Helsinki.

### SMME

Soluble mucins were enriched from MEC and NSGs in the manner reported in previous studies^25,26^. SMME membranes were prepared as previously described^14^. The SMME membranes (separation length, 6 cm) were wetted in methanol and then transferred into a running buffer (0.1 M pyridine-formic acid buffer, pH 4.0). After equilibration for 30 min with gentle shaking, the membranes were subjected to electrophoresis. The enriched solutions containing soluble mucins and porcine gastric mucin (PGM) (type III, partially purified) (Sigma-Aldrich, St. Louis, MO, USA) were spotted at 1.5 cm from the edge of the membrane at the negative node. Electrophoresis was performed by using a membrane electrophoresis chamber (EPC105AA; Advantec, Tokyo, Japan) in constant-current mode at 1.0 mA/cm for 30 min.

### Lectin blotting

The glycoproteins on the SMME membranes were stained with lectins according to the immunostaining method previously reported^26–28^. Briefly, the electrophoresed membranes were immersed in acetone for 30 min, followed by heating at 150 °C for 5 min^29,30^. The membranes were blocked by immersing in 5% BSA or 5% PGM in phosphate-buffered saline (PBS) containing 0.05% Tween-20 (PBS-T) for 1 h and then incubated with biotin-labeled lectins that are, *Sambucus sieboldiana* agglutinin (SSA) (J-CHEMICAL, Inc. Tokyo, Japan), *Aleuria aurantia lectin* (AAL) (J-CHEMICAL), *Maackia amrensis* lectin II (MAL-II) (Vector Laboratories, Inc. Burlingame, California, USA) and N-terminal domain of BC2L-C lectin derived from *Burkholderia cenocepacia* (BC2LCN) (FUJIFILM Wako Pure Chemical Corporation, Osaka, Japan) in PBS-T (1/500) for 1 h at room temperature, respectively. Lectin binding was visualised by using the streptavidin–horseradish peroxidase (HRP) (Vector) conjugate and a chemiluminescence reagent (Western Lightning Plus-ECL; PerkinElmer, Boston, MA, USA). Chemiluminescence images were obtained by using ChemiDoc XRS (Bio-Rad, Hercules, CA, USA) and visualized using Image J v1.53e.

### Immunohistochemistry in FFPE sections

All MEC samples were fixed in 10% buffered formalin, processed and embedded in paraffin according to routine procedures. FFPE of MEC specimens were cut into 3–4 μm sections, warmed overnight at 60 °C, deparaffinised in xylene and rehydrated through a graded alcohol series. For antigen retrieval, the sections were microwave-treated in 10 mM citrate buffer (pH 6.0) for 10 min and washed two times with PBS. The cooled sections were incubated for 5 min in DAKO REAL Peroxidase-Blocking Solution (DAKO, Glostrup, Denmark) to deactivate endogenous peroxidases and washed two times with PBS. Nonspecific binding was blocked with 1% bovine serum albumin in 10 mM PBS for 1 h. The sections were incubated with anti-MUC1 antibody (E29) (Abcam Ltd., Cambridge, MA, USA), anti-sialyl-Tn antibody (Abcam) and MAL-II in PBS (diluted 1/50) for 90 min at room temperature and washed three times with PBS. The sections were incubated with secondary antibody in PBS (diluted 1/500) and washed three times with PBS. HRP activity was visualised by using the DAB Substrate kit (Abcam). Finally, the sections were counterstained with haematoxylin, washed, dehydrated and mounted. Negative controls were performed by omitting the primary antibody. The serial sections were also stained with haematoxylin and eosin (HE) to assess tumour histology. The tumours and surrounding NSGs were considered positive when showing ≥ 5% positive neoplastic cells.

### ISH in FFPE sections

All MEC samples were fixed in 10% buffered formalin, processed and embedded in paraffin according to routine procedures. ISH of *C2GnT-1* was commissioned to Genostaff Co., Ltd. (Tokyo, Japan). Briefly, the FFPE sections were deparaffinised and rehydrated through an ethanol series and PBS. Then, the sections were fixed with 10% neutral-buffered formalin, treated with 4–10 µg/ml proteinase K (Wako Pure Chemical Industries Ltd.) and placed within a Coplin jar containing 1 × saline-sodium citrate (SSC). Hybridisation was performed with probes (250 ng/ml) in G-Hybo-L (Genostaff) for 16 h at 60 °C, and then the sections were washed three times with 50% formamide in 2 × SSC for 30 min at 50 °C, and five times in 0.1% Tween 20 in Tris-buffered saline (TBS-T) at room temperature. After blocking with 1 × G-Block (Genostaff) for 15 min at room temperature, the sections were incubated with anti-digoxigenin-conjugated alkaline phosphatase (Anti-Digoxigenin AP, Fab fragments, Roche Diagnostics GmbH, Mannheim, Germany) diluted 1:2000 with G-Block (diluted 1/50) in TBS-T for 1 h at room temperature. The sections were washed twice in TBS-T and then incubated in 100 mM Tris–HCl buffer (pH 9.5) containing 100 mM NaCl, 50 mM MgCl_2_ and 0.1% Tween 20. The presence of C2GnT-1 mRNA was visualised with NBT/BCIP solution (Sigma-Aldrich) and then washed in PBS. The sections were counterstained with Kernechtrot solution (Muto pure chemicals Co., Ltd. Tokyo, Japan) and mounted with G-Mount (Genostaff). The Images were visualized using Image J v1.53e. The tumours and surrounding NSGs were considered positive when showing ≥ 5% positive neoplastic cells.

## Conclusion

MUC1 modified with α2,3-linked sialic acid-containing core-2 *O-*glycans in MEC was distinctively expressed in mucous cells and non-mucous cells. MUC1 modified with these glycans deserves further study as a potential diagnostic marker of MEC.

## Supplementary Information

Supplementary Information.

## Data Availability

The data that support the fundings of this study is available from the corresponding author upon request.
