# Supplementary material for: Expression and localisation of MUC1 modified with sialylated core-2 O-glycans in mucoepidermoid carcinoma
Source: Sci Rep. 2023 Apr 8;13:5752. doi: 10.1038/s41598-023-32597-2 (PMC10082819; doi:10.1038/s41598-023-32597-2)
Supplement: Supplementary file 1 — Supplementary Information. [file 41598_2023_32597_MOESM1_ESM.pdf]

Expression and localization of MUC1 modified with sialylated core 2 *O*-glycans in mucoepidermoid carcinoma

Supplemental Table and Figures

Table S1. Summary of MUC1, sialyl-Tn, MAL-Ⅱ and C2GnT-1 expression in the mucoepidermoid carcinoma lesion and the surrounding normal salivary tissue in each sample.

		MUC1			sialyl-Tn			MAL-II			C2GnT-1		
Sample number		1	2	3	1	2	3	1	2	3	1	2	3
Normal salivary glands	Mucous acini	−	−	−	−	−	−	＋	＋	＋	−	−	−
	Serous acini	−	−	−	−	−	−	＋	＋	＋	−	−	−
	Ducts	＋	−	＋	−	−	−	＋	＋	＋	−	−	−
MEC tissue	Mucous cells	＋	＋	＋	−	＋	−	＋	＋	＋	＋	＋	＋
	Non-mucous cells	＋	＋	＋	−	＋	−	＋	＋	＋	＋	＋	＋

＋：Positive, －: Negative

Fig. S1. The original, unprocessed versions of full-length lectin blotting of the SMME membranes (separation length, 6 cm) separating salivary gland homogenates for Fig. 1. (A) MA L-Ⅱ; (B) SSA; (C) BC2LCN; (D) AAL.

Fig. S2. Expression patterns of MUC1, Sialy-Tn, sialoglycans and C2GnT-1 in surrounding normal salivary gland of sample 1. The representative images (original magnification, ×400) of H&E staining (A), staining with anti-MUC1 antibody (B), staining with anti-Sialyl-Tn antibody (C), staining with MAL-Ⅱ (D), and ISH of C2GnT-1 (E) are shown. Scale bars indicate 100 µm.

Fig. S3. Expression patterns of MUC1, Sialy-Tn, sialoglycans and C2GnT-1 in MEC of sample 1. The representative images (original magnification, ×400) of H&E staining (A), staining with anti-MUC1 antibody (B), staining with anti-Sialyl-Tn antibody (C), staining with MAL-Ⅱ (D), and ISH of C2GnT-1 (E) are shown. Scale bars indicate 100 µm.

Fig. S4. Expression patterns of MUC1, Sialy-Tn, sialoglycans and C2GnT-1 in surrounding normal salivary gland of sample 2. The representative images (original magnification, ×400) of H&E staining (A), staining with anti-MUC1 antibody (B), staining with anti-Sialyl-Tn antibody (C), staining with MAL-Ⅱ (D), and ISH of C2GnT-1 (E) are shown. Scale bars indicate 100 µm.

Fig. S5. Expression patterns of MUC1, Sialy-Tn, sialoglycans and C2GnT-1 in MEC of sample 2. The representative images (original magnification, ×400) of H&E staining (A), staining with anti-MUC1 antibody (B), staining with anti-Sialyl-Tn antibody (C), staining with MAL-Ⅱ (D), and ISH of C2GnT-1 (E) are shown. Scale bars indicate 100 µm.

Fig. S6. Expression patterns of MUC1, Sialy-Tn, sialoglycans and C2GnT-1 in surrounding normal salivary gland of sample 3. The representative images (original magnification, ×400) of H&E staining (A), staining with anti-MUC1 antibody (B), staining with anti-Sialyl-Tn antibody (C), staining with MAL-Ⅱ (D), and ISH of C2GnT-1 (E) are shown. Scale bars indicate 100 µm.

Fig. S7. Expression patterns of MUC1, Sialy-Tn, sialoglycans and C2GnT-1 in MEC of sample 3. The representative images (original magnification, ×400) of H&E staining (A), staining with anti-MUC1 antibody (B), staining with anti-Sialyl-Tn antibody (C), staining with MAL-Ⅱ (D), and ISH of C2GnT-1 (E) are shown.　Scale bars indicate 100 µm.

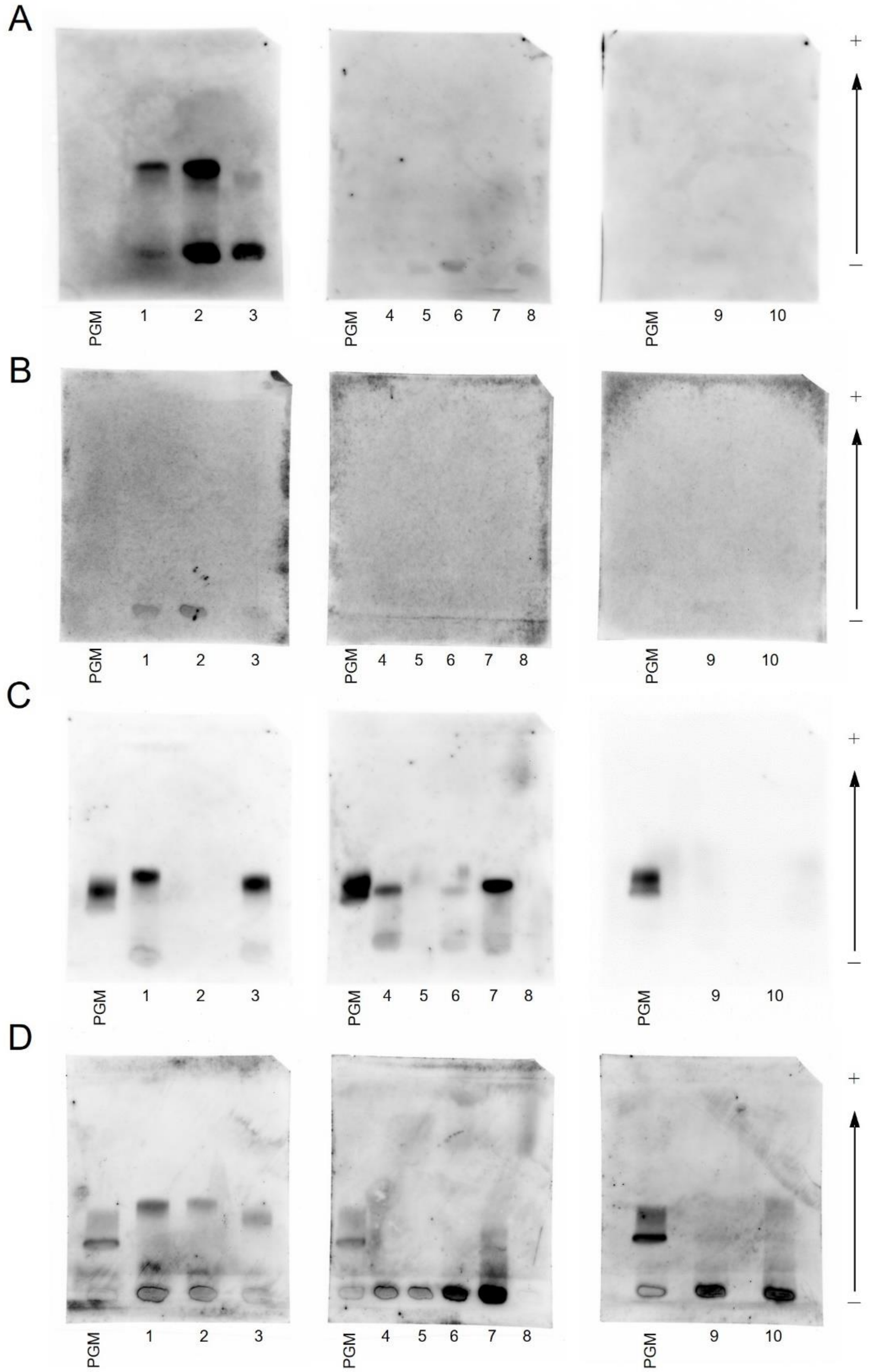
A
PGM
1
2
3
PGM
4
5
6
7
8
PGM
9
10
+
–
B
PGM
1
2
3
PGM
4
5
6
7
8
PGM
9
10
+
–
C
PGM
1
2
3
PGM
4
5
6
7
8
PGM
9
10
+
–
D
PGM
1
2
3
PGM
4
5
6
7
8
PGM
9
10
+
–

Fig. S1

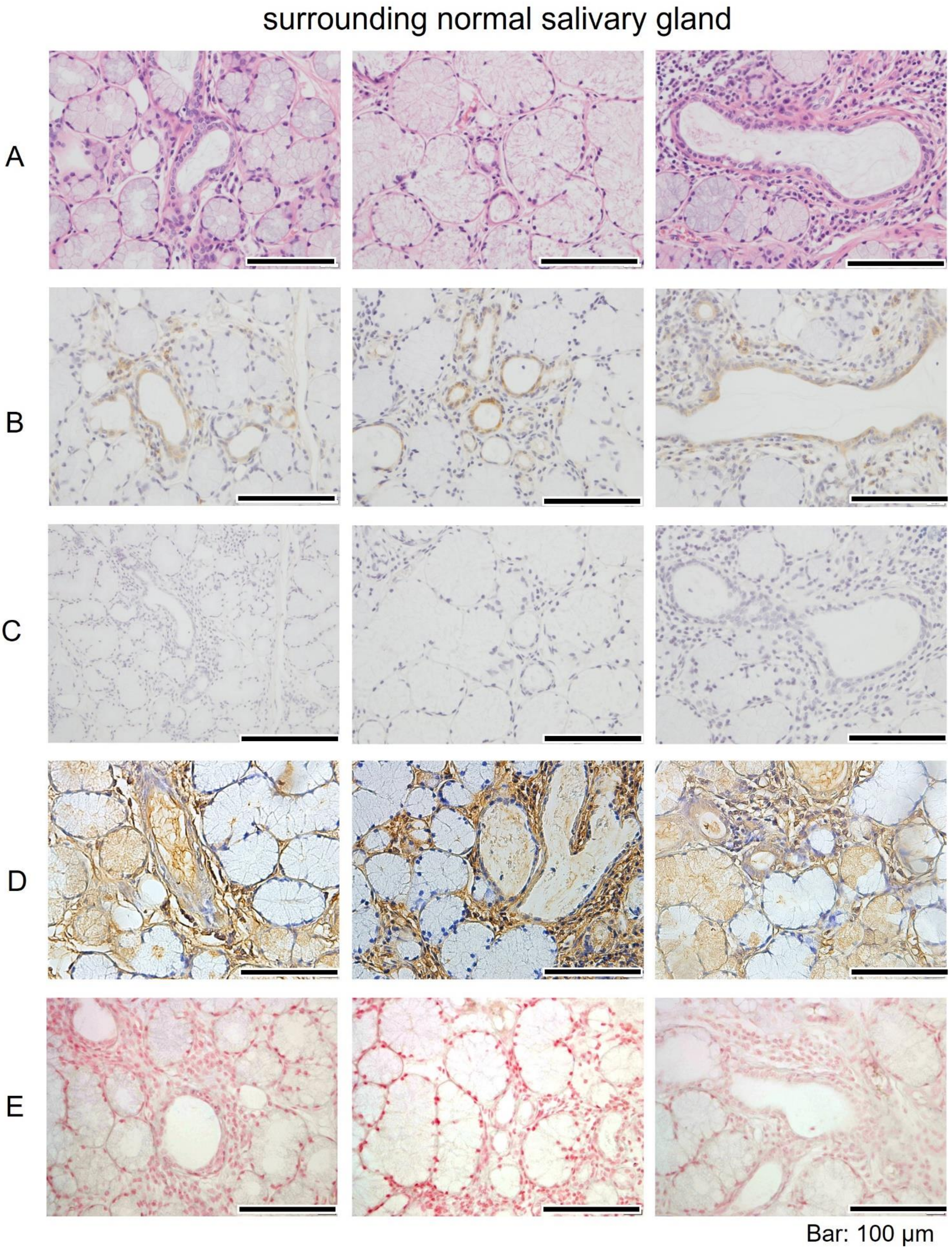

Fig. S2

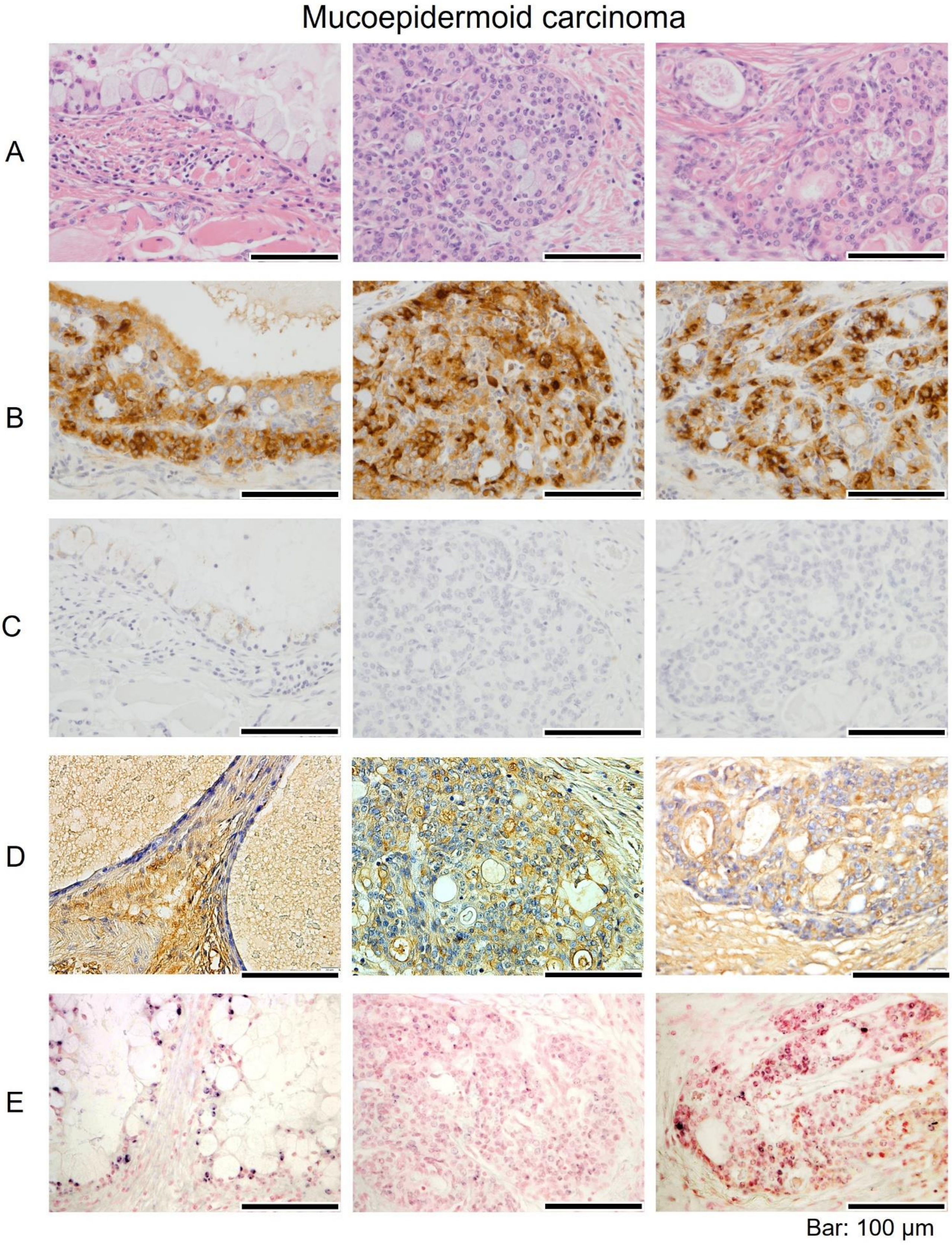

Fig. S3

surrounding normal salivary gland

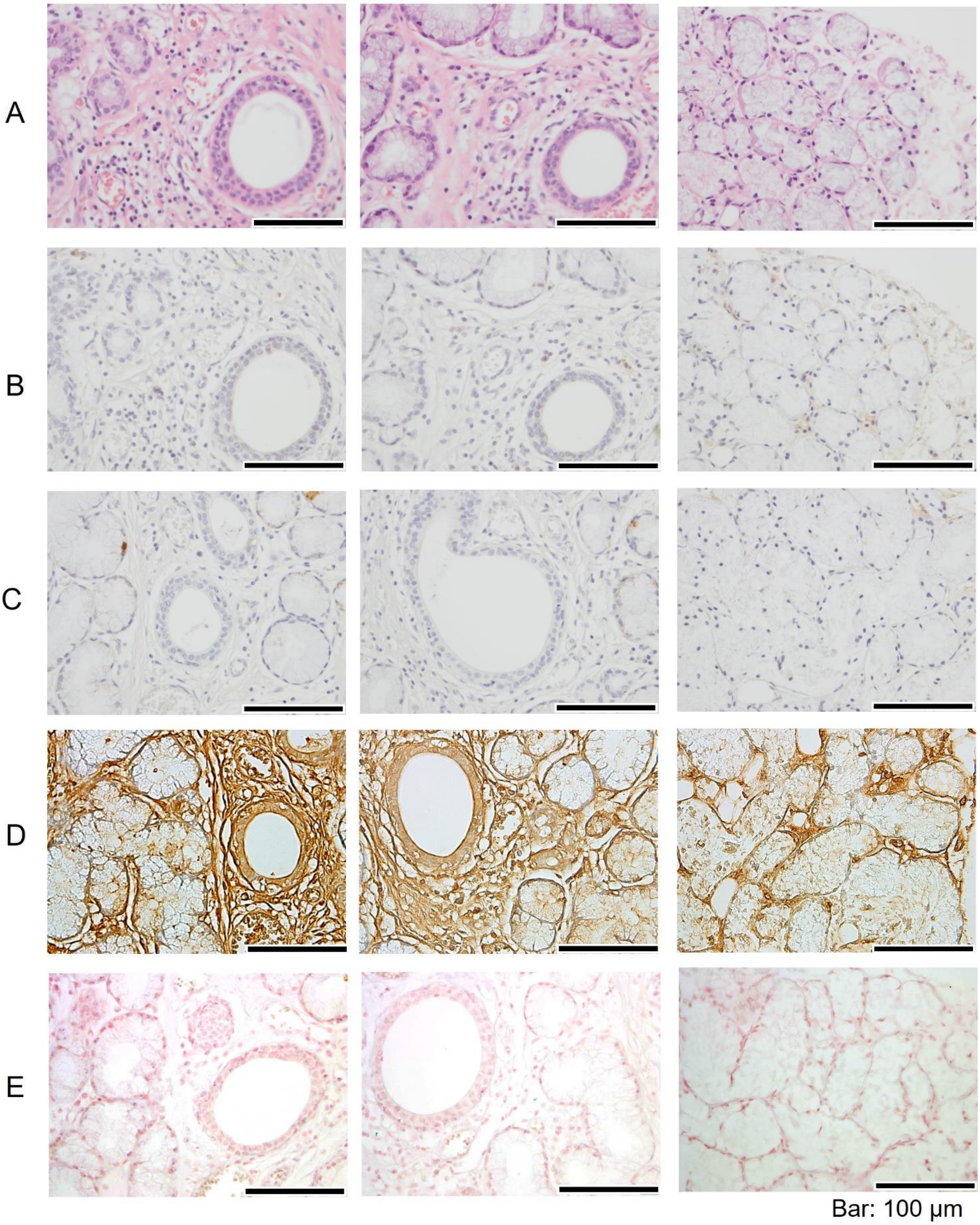

Fig. S4

Mucoepidermoid carcinoma

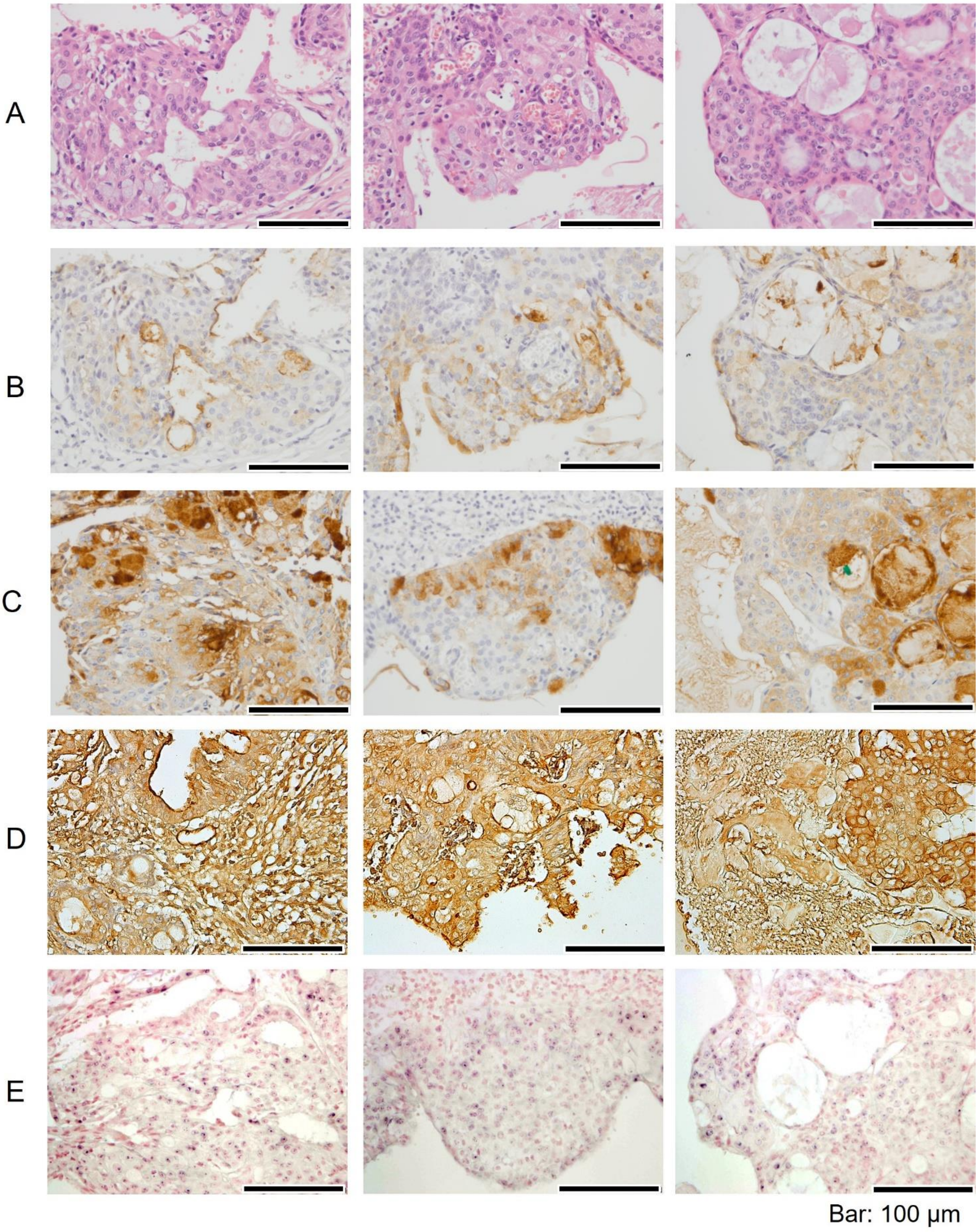

Fig. S5

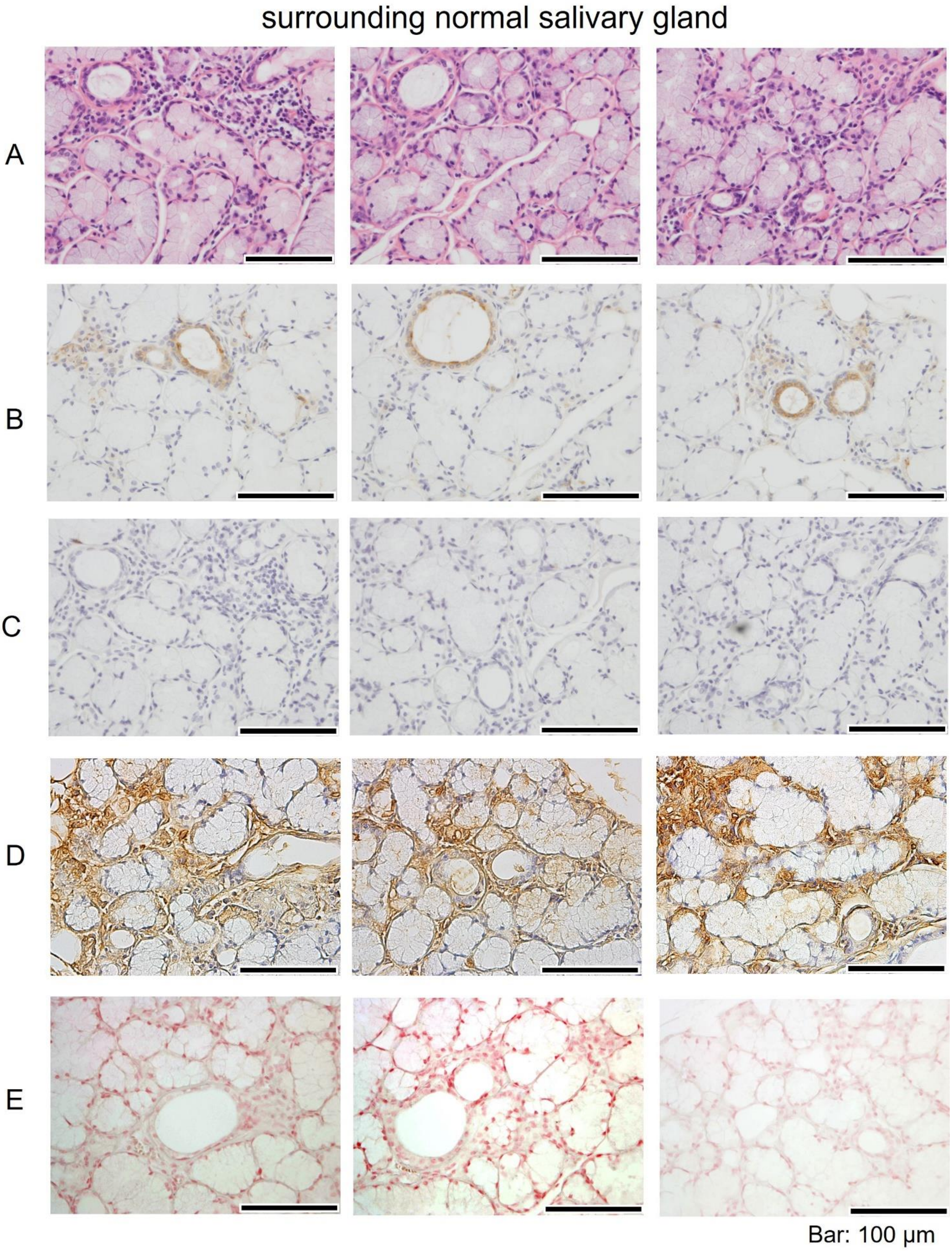

Fig. S6

Mucoepidermoid carcinoma

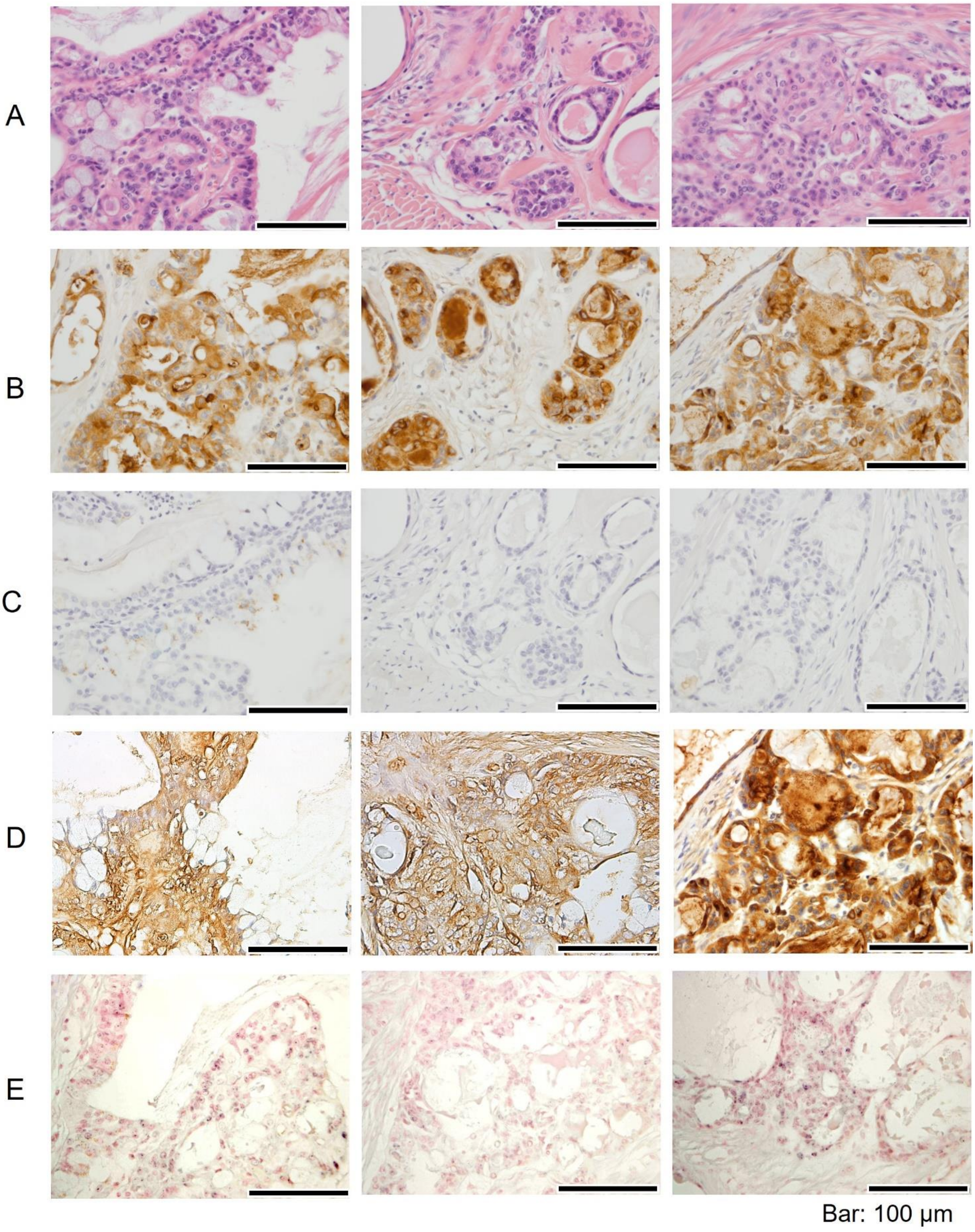

Fig. S7
